# HER2 Positivity in Histological Subtypes of Salivary Gland Carcinoma: A Systematic Review and Meta-Analysis

**DOI:** 10.3389/fonc.2021.693394

**Published:** 2021-06-24

**Authors:** Kristian Egebjerg, Cecilie Dupont Harwood, Nina Claire Woller, Claus Andrup Kristensen, Morten Mau-Sørensen

**Affiliations:** ^1^ Department of Oncology, Copenhagen University Hospital, Rigshospitalet, Copenhagen, Denmark; ^2^ Department of Otorhinolaryngology, Head & Neck Surgery and Audiology, Copenhagen University Hospital, Rigshospitalet, Copenhagen, Denmark; ^3^ Department of Pathology, Copenhagen University Hospital, Rigshospitalet, Copenhagen, Denmark

**Keywords:** HER2, salivary gland (S.G) tumors, ERBB2, salivary duct carcinoma, prevalence

## Abstract

**Background:**

HER2 aberrations in salivary gland carcinomas (SGC) as well as benefit of HER2 directed therapy have been reported in small studies. However, reliable estimates of the prevalence of HER2 positivity in SGC and its various histological subtypes are lacking.

**Objective:**

To assess the prevalence of HER2 positivity in histological subtypes of salivary gland carcinomas (SGC).

**Methods:**

Studies were identified by a systematic review of the literature. Data on *in situ* hybridization (ISH) and immunohistochemistry (IHC) were extracted to derive pooled prevalence estimates calculated by a random effects model. Characteristics of the studies were extracted for subgroup analysis.

**Results:**

Fifty studies including 3372 patients were identified, providing data on sixteen histological subtypes. Based on the meta-analysis, the estimated prevalence of HER2 positivity were 43% (95% CI: 36% – 51%) in salivary duct carcinoma (SDC), 39% (95% CI: 32% – 45%) in carcinoma ex pleomorphic adenoma (CEP), 17% (95% CI: 7.5% – 33%) in squamous cell carcinoma (SCC), 13% (95% CI: 7.6% – 21%) in adenocarcinoma NOS (ADC), 6.7% (95% CI: 0.17%-32%) in poorly differentiated carcinoma, 5.5% (95% CI: 2.9% – 9.6%) in mucoepidermoid carcinoma, 4.3% (95% CI: 1.4% – 13%) in myoepithelial carcinoma, 1.8% (95% CI: 0.04%-9.6%) in epithelial-myoepithelial carcinoma, 0.45% (95% CI: 0.0097% – 18%) in acinic cell carcinoma and 0.15% (0.037% – 5.4%) in adenoid cystic carcinoma. Estimates for five additional subtypes were assessed.

**Conclusion:**

Prevalence of HER 2 positivity in SGC varies greatly based on histological subtype, with SDC, CEP, SCC, and ADC displaying the highest rates.

## Introduction

Salivary gland carcinomas (SGC) are relatively rare tumors with an annual worldwide incidence of 0.07% corresponding to 52,799 cases each year according to the Global Cancer Observatory (1). The most recent WHO classification divide SGC into 21 histological subtypes (2). The incidence of the most common histopathological subtypes vary between countries, but mucoepidermoid carcinoma is the most prevalent subtype making up 12%-29% of the total cases, adenoid cystic carcinomas accounts for 10%-22%, acinic cell carcinoma for 8%-14%, while salivary duct carcinomas (SDC) only account for 5%-10%. SDC represents the most aggressive type (3–5). The prognosis of metastasizing SGC remains poor, and response rates to chemotherapy are modest (4). Consequently, oncologists and patients alike are faced with a clear unmet medical need for improvements in the treatment of this disease (6).

HER2 is a human epidermal receptor 2 tyrosine kinase of the epidermal growth factor receptor (EGFR) class coded by an oncogene *ERBB2* located on chromosome 17. HER2 is overexpressed in various subtypes of SGC, but clinical trials on HER2 targeted therapy with trastuzumab or lapatanib without chemotherapy in SGC have failed to show significant clinical benefit, maybe because only a subset of the lapatinib treated patients harbored tumors with HER2 overexpression (7, 8). However, a Japanese study combining trastuzumab and docetaxel found an overall response rate of 70% in patients with HER2 positive SDC defined as IHC3+ or gene amplification by FISH (9). Recently, novel HER2 targeted therapies such as ado-trastuzumab emtansine and combinations of trastuzumab and pertuzumab have reached relevant response rate of 90% and 60%, respectively (10, 11).

HER2 protein overexpression is measured semi-quantitatively by immunohistochemistry (IHC) and gene amplification is measured by fluorescence/silver/dual *in situ* hybridization (FISH, SISH and DISH). Various scoring systems exist for other cancer types, such as breast carcinoma and gastric esophageal adenocarcinoma (12, 13). Although specific criteria for SGC have been proposed, the breast cancer carcinoma criteria are the most commonly used for scoring HER2 expression in SGC (14). This is partially due to morphological similarities to invasive ductal carcinoma of the breast and molecular resemblance with apocrine breast cancer and because studies validating HER2 scoring systems in SGC are lacking (15).

HER2 overexpression or gene amplification seems to be a prerequisite for response to trastuzumab. Currently there is no systematic review or meta-analysis investigating the prevalence of HER2 in SGC. The aims of this review and meta-analysis are to evaluate the literature and provide prevalence estimates for HER2 in various histological subtypes of SGC.

## Methods

PRISMA Reporting guidelines were used.

### Eligibility

Inclusion Criteria: Only studies examining human SGC tissue were included. Studies allowed were clinical trials, prospective and retrospective observational studies provided the study population was not a preselected HER2 positive cohort. HER2 status had to be evaluated by *either* IHC reporting semi-quantitative scores of 0, 1+, 2+, 3+ *or* quantitative ratios of HER2 gene copy number relative to chromosome 17 by ISH *or* by both IHC and ISH. Studies reporting HER2 status dichotomously (HER2 positive/negative) using the above mentioned semi-quantitative or quantitative data were eligible.

Exclusion criteria: Studies not listing which quantitative scoring methods of IHC 0 to 3+ or ISH were used to define HER2 positivity were not included. Studies not discriminating between histological subtype and HER2 status were not included. If the same dataset of patients was reported by the same author in two different publications only the newest was included. Studies reported in languages other than English, unpublished studies, case studies, conference abstracts, cell line and animal studies were all considered ineligible.

Rationale for criteria: The above-mentioned inclusion criteria were chosen to gather sufficient data to evaluate HER2 positivity in specific histological subtypes, and to assess whether criteria of HER2 positivity affect the prevalence estimates.

### Identifying Studies

PubMed, Embase, Web of Science were searched up to September 19^th^, 2020 using the search string ((salivary gland tumor[Title/Abstract] OR carcinoma of the salivary gland[Title/Abstract] OR salivary gland cancer[Title/Abstract])) AND (HER2 or c-ERB2). The search syntaxes were adapted to those used by each respective search engine. All time periods were included. Exact search-syntax used for each search engine can be seen in **Supplemental S2**. No limitations were set regarding the date of coverage. In addition, hand searching of references list of obtained articles was conducted.

### Study Selection Process

Titles were identified by the above-mentioned search strategy, screened and assessed for inclusion in the final meta-analysis independently by KE and CDH. Discrepancies were solved by consensus. A full list of texts screened but not included as well as the reason for exclusion is listed in **Supplement S3**.

### Risk of Bias in the Individual Studies and Across Studies

The eligibility criteria were designed to minimize risk of bias – especially selection bias, across studies.

As the studies included are observational and not randomized controlled trials or interventional in nature, risks of bias were assessed using recommendations from COSMOS-E (Conducting Systematic Reviews and Meta-Analyses of Observational Studies of Etiology) (16).

Information bias was assessed by registering methods potentially affecting how frequently the outcome were registered: Prospectively collected or archival samples, HER2 positivity criteria, IHC assay and ISH probe type. The latter were also treated as confounders together with Geographic Region.

### Data Items and Collection

A data extraction form was used to extract equivalent information from each paper. First author, published year, geographical region, prospectively collected or archival samples, HER2 positivity criteria, IHC assay, ISH probe type and ISH type: FISH, DISH, SISH. In addition, number of patients with each histological subtype and number of HER2 positive patients as well as data on, IHC0, IHC1+, IHC2+, IHC3+, and HER2 amplification were collected.

### Specification of Endpoints

The following endpoints were predefined:

The primary endpoint was HER2 positivity for each SGC histological subtype. Specific IHC data (0, 1+, 2+, 3+) and gene amplification status was extracted when possible. During the data collection it became clear that this specific data was only available for SDC.

### Analysis and Statistics and Synthesis Methods

Studies were included in each respective meta-analysis depending on the available data. Meta-analyses were conducted using a random effects model. The Wilson score interval method was used to calculate confidence intervals. Maximum likelihood estimator was used to estimate between study variance tau^2^ with the inverse variance method. Generalized linear mixed models were used for pooled prevalence estimates, forest plots were created and sorted based on number of patients included. Whenever sufficient data were available, subgrouping based on HER2 definition was plotted, and subgroup analysis based on probe, assay, geographical region was also conducted.

A threshold of n>60 patients was chosen for each tissue type to conduct meta-analysis, as we believe a lower number of patients would not yield a meaningful meta-analysis.

The Clopper-Pearson interval was used to calculate 95% confidence intervals in tissue types not eligible for inclusion in meta-analysis.

R version 4.0.0 and package meta was used.

### HER2 Positivity

Various criteria were employed by studies to characterize tumor tissue as “HER2 positive”, and each study was labelled according to criteria employed. When data on both IHC and FISH status were reported, IHC2+ confirmed by gene amplification or IHC3+ was preferentially defined as HER2 positive.

### IHC and FISH Prevalence Among SDC

Data for SDC, both *de novo* and carcinoma ex pleomorphic adenoma were sufficient to conduct analysis for specific IHC status sand gene amplification. Two studies (17, 18) reported combined estimates of IHC0 and IHC1+; this estimate was divided by two and each half was included in the IHC0 and IHC1+ analysis respectively.

## Results

**Figure 1:**
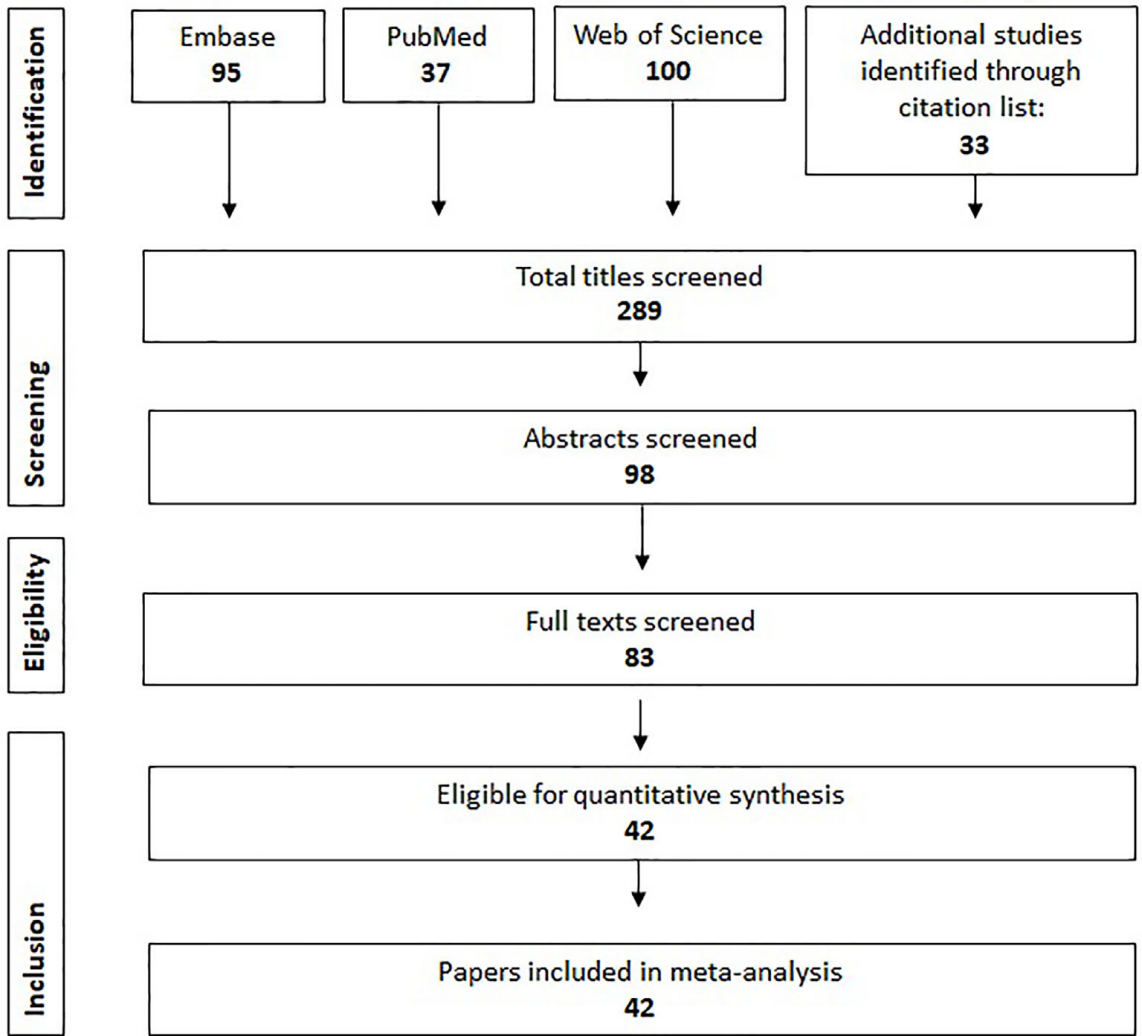
Flowchart describing the methodology of article selection.

By the indicated method of study selection (**Figure 1**), 50 studies were identified including a total number of 3,372 patients to study the prevalence of HER2 positivity in SGC (**Table 1**, full characteristics of studies, **Supplemental S1**). Archival tissue was used in all studies except one; in this study information about tissue sampling was not available. Nineteen studies were conducted in Europe, 12 studies in the Americas, eight in Asia, two in Oceania and one study conducted in both Europe and the Americas. The following criteria were used in the studies included to define HER2 positivity: (1) IHC2+ or IHC3+, (2) IHC3+, (3) IHC2+ and HER2 amplification assessed by ISH or IHC3+, (4) IHC2+ or IHC3+ or HER2 amplification assessed by ISH, (5) IHC3+ or HER2 amplification assessed by ISH, (6) HER2 amplification assessed by ISH, (7) IHC2+ and ISH or IHC3 and ISH.

**Table 1:** Studies included in the meta-analysis.

First Author	Year	Geographic Region	Criteria for HER2 Positivity Criteria	Number of patients
Khan (19)	2001	America	IHC3	29
Skálová (20)	2001	Europe	IHC2 or IHC3	29
Dori (21)	2002	Asia	IHC3	32
Skalova (22)	2003	Europe	IHC2 and ISH or IHC3	11
Glisson (17)	2004	America	IHC2 or IHC3	136
Weed (23)	2004	America	IHC2 or IHC3	28
Di Palma (24)	2005	Europe	IHC3	11
Jaehne (25)	2005	Europe	IHC3	34
Cornolti (26)	2007	Europe	IHC3 or ISH	13
Nabili (27)	2007	America	IHC3	7
Tapia (28)	2007	Europe	IHC2 and ISH or IHC3	12
Williams (29)	2007	America	IHC3	59
Ettl (30)	2008	Europe	IHC2 or IHC3	91
Shang (31)	2008	Asia	IHC2 or IHC3	46
Locati (32)	2009	Europe	IHC2 and ISH or IHC3	123
Luukkaa (33)	2010	Europe and America	ISH	11
Williams (34)	2010	America	IHC2 and ISH or IHC3	66
Clauditz (35)	2011	Europe	IHC3 or ISH	915
Di Palma (36)	2012	Europe	IHC2 and ISH or IHC3	42
Ettl (37)	2012	Europe	IHC3	235
Hashimoto (38)	2012	Asia	IHC2 and ISH or IHC3	31
Suzuki (39)	2012	Asia	IHC2 or IHC3	45
Cros (40)	2013	Europe	IHC3	28
Nakano (41)	2013	Asia	IHC2 or IHC3	31
Nardi (42)	2013	America	ISH	19
Kondo (43)	2014	Asia	IHC2 and ISH or IHC3	13
Masubuchi (44)	2014	Asia	ICH3 and ISH	32
Han (45)	2015	Asia	IHC2 and ISH or IHC3	25
Jakob (46)	2015	America	IHC2 or IHC3	16
Nishijima (47)	2015	Asia	IHC2 or IHC3	50
Kusafuka (48)	2016	Asia	IHC2 and ISH or IHC3	9
Locati (49)	2016	Europe	IHC2 and ISH or IHC3	11
Lemound (50)	2016	Europe	IHC3 or ISH	37
Luk (51)	2016	Oceania	IHC2 and ISH or IHC3	23
Hashimoto (52)	2017	Asia	IHC2 and ISH or IHC3	221
Khoo (53)	2017	Oceania	ISH	15
Locati (54)	2017	Europe	IHC2 and ISH or IHC3	28
Takase (55)	2017	Asia	ICH3 or ISH	151
Andreasen (56)	2018	Europe	IHC2 and ISH or IHC3 and ISH	73
Beck (57)	2018	Europe	IHC2 and ISH or IHC3	15
Boon (58)	2018	Europe	IHC2 and ISH or IHC3	153
Kanazawa (59)	2018	Asia	IHC3	34
Ryu (60)	2018	Asia	ISH	28
Gargano (61)	2019	America	IHC3	28
Liang (62)	2019	America	IHC3	86
Santana (63)	2019	Europe	IHC2 and ISH or IHC3	24
Szewczyk (64)	2019	Europe	IHC2 and ISH or IHC3	115
Villeplet (18)	2019	Europe	IHC2 and ISH or IHC3	36
Chatzopoulos (14)	2020	America	IHC2 and ISH or IHC3	32
Hsieh (65)	2020	Europe	IHC2 and ISH or IHC3	33

### Salivary Duct Carcinoma: IHC

**Figure 2:**
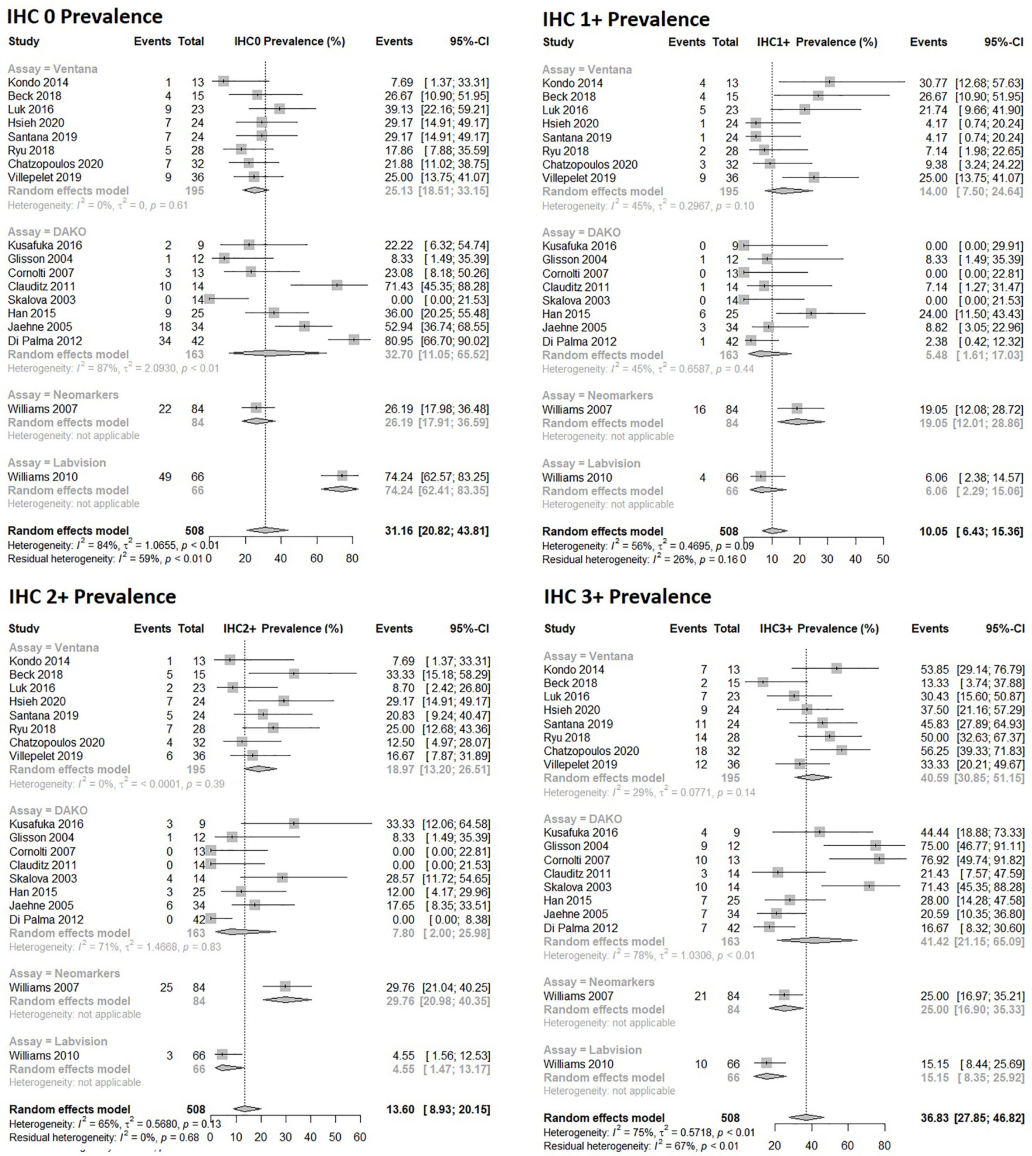
Forrest plot of prevalence estimates for HER2 protein expression assessed by IHC in salivary duct carcinomas.

Eighteen studies were included in the analysis of prevalence of protein expression as assessed by IHC in SDC patients. The estimated prevalence of HER2 scores of IHC0 was 31% (95% CI: 21% - 44%), IHC1+ 10% (95% CI: 6.4% – 15%), IHC2+ 14% (95% CI: 8.9%-20%), and IHC3+ 37% (95% CI: 28%-47%) as presented in **Figure 2**. There was significant (p<0.01) and marked heterogeneity in the IHC0 and IHC3+ data with I^2^ of 59% and 67%, respectively, but no significant heterogeneity in the IHC1+ and IHC2+ data. There was significant difference between assays used for all four IHC HER2 scores, for further information see **Supplemental S4**.

### Salivary Duct Carcinoma: HER2 Gene Amplification

**Figure 3:**
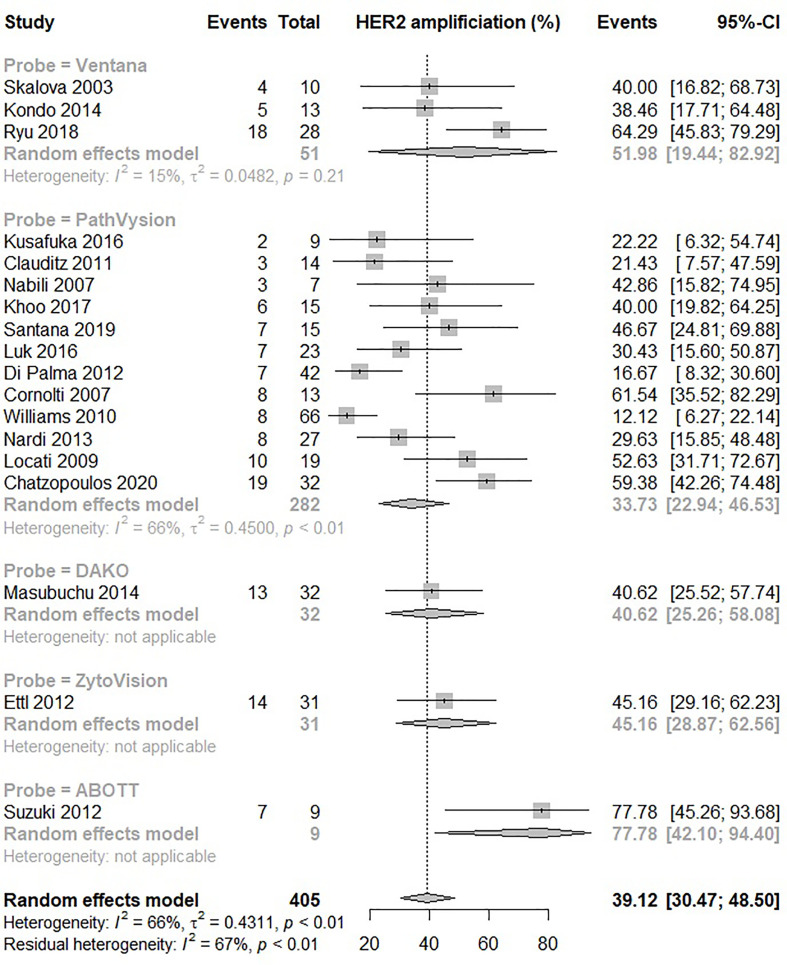
Forrest plot of HER2 gene amplification rate in SDC assessed by in situ hybridization.

Eighteen studies were included in the analysis. HER2 amplification rate in SDC was found to be 39% (95% CI: 31-49) as shown in **Figure 3**. There was significant (p<0.01) and marked (I^2^ 66%) heterogeneity between studies. There was no significant difference in the estimated prevalence between studies applying various probes (p=0.12).

### Salivary Duct Carcinoma: HER2 Positivity

**Figure 4:**
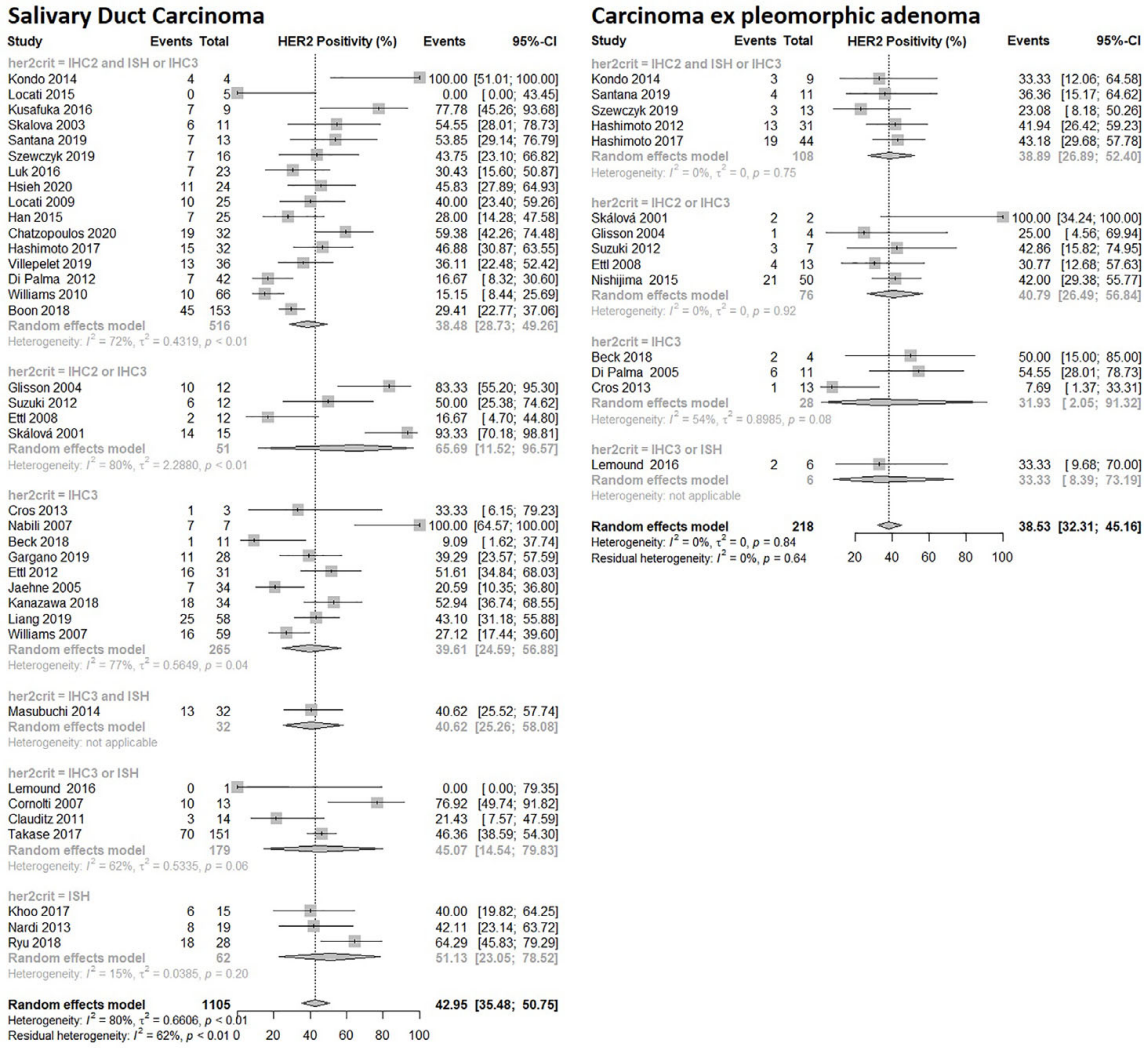
Forrest plot of HER2 Prevalence among SGC subtypes: Salivary Duct Carcinoma and Carcinoma ex pleomorphic adenoma.

Thirty-seven studies with a total of 1,105 patients were included in the random effects model. The model predicted a prevalence of HER2 positivity in SDC patients to be 43% (95% CI: 36% – 51%) depicted in **Figure 4**. The heterogeneity between the studies was significant p<0.01, and substantial, I^2 =^ 80%. There was significant difference between assays p=0.0017, although the differences seemed to level off for the most commonly used assays: Prevalence of 46% (95% CI: 32%- 62%) and 44% (95% CI: 36%- 53%) were estimated for 19 and 11 studies using DAKO and Ventana assays, respectively. Prevalence of less commonly used assays are shown in **Supplemental S5**.

There were no differences in the prevalence between studies using varying criteria for HER2 positivity (p=0.61) or conducted in different geographical regions (p=0.16).

### Carcinoma Ex Pleomorphic Adenoma (CEP): HER2 Positivity

Fourteen studies were included in the random effects model with a total of 218 patients. The model predicted a prevalence of HER2 positivity in CEP patients to be 39% (95% CI: 32% – 45%) depicted in **Figure 4**. The heterogeneity between studies was not significant. There were no statistical differences based on the applied criteria for HER2 positivity (p=0.95), used assays (p=0.46) or the geographical regions (p=0.48).

### Adenocarcinoma NOS (ADC NOS): HER2 Positivity

**Figure 5:**
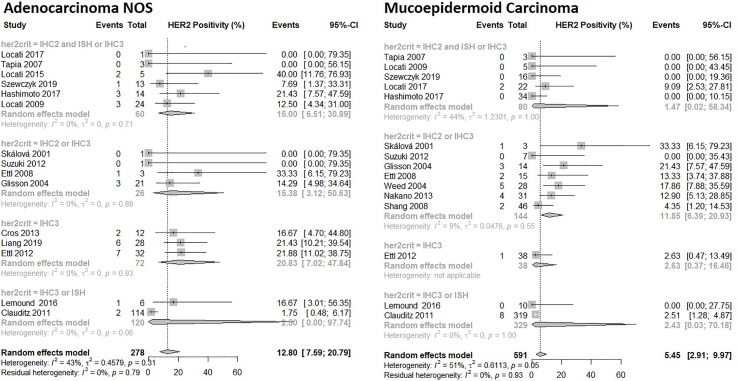
Forrest plot of HER2 Prevalence among SGC subtypes: Adenocarcinoma NOS and Mucoepidermoid Carcinoma.

Fifteen studies were included in the random effects model with a total of 275 patients. The model predicted a prevalence of HER2 positivity in ADC NOS tumors of 13% (95% CI: 7.6% – 21%) as shown in **Figure 5**. The heterogeneity between studies was not significant. The prevalence were significantly different when comparing studies using different criteria for HER2 positivity (p=0.0052). However, the estimated prevalence was higher in those studies using the narrowest criteria for HER2 positivity. Neither geographical region (p=0.47) nor assay (p=0.30) used was associated with differences in prevalence.

### Mucoepidermoid Carcinoma: HER2 Positivity

Fifteen studies with a total of 591 patients were included in the random effects model. The model predicted a prevalence of HER2 positivity in mucoepidermoid carcinoma patients to be 5.5% (95% CI: 2.9% – 9.6%) as seen in **Figure 5**. The heterogeneity between studies was moderate I^2 =^ 51% and statistically significant p=0.050. There were significant differences in the prevalence between subgroups based on criteria for HER2 positivity (p=0.0014) and geographical region (p=0.0002). The broadest criteria defining HER2 positivity as IHC2+ and IHC3+ reached prevalence estimates of 12% (95% CI: 6.4% -21%). Two American studies resulted in prevalence estimates by the random effect model of 19% (95% CI: 0.16% – 97%), four Asian studies in prevalence estimates of 4.1 (95% CI: 0.41%-30%) and nine studies from Europe in prevalence estimates of 3.3% (95% CI: 1.8% - 5.9). There was no significant difference between assays used (p=0.56).

### Myoepithelial Carcinoma: HER2 Positivity

**Figure 6:**
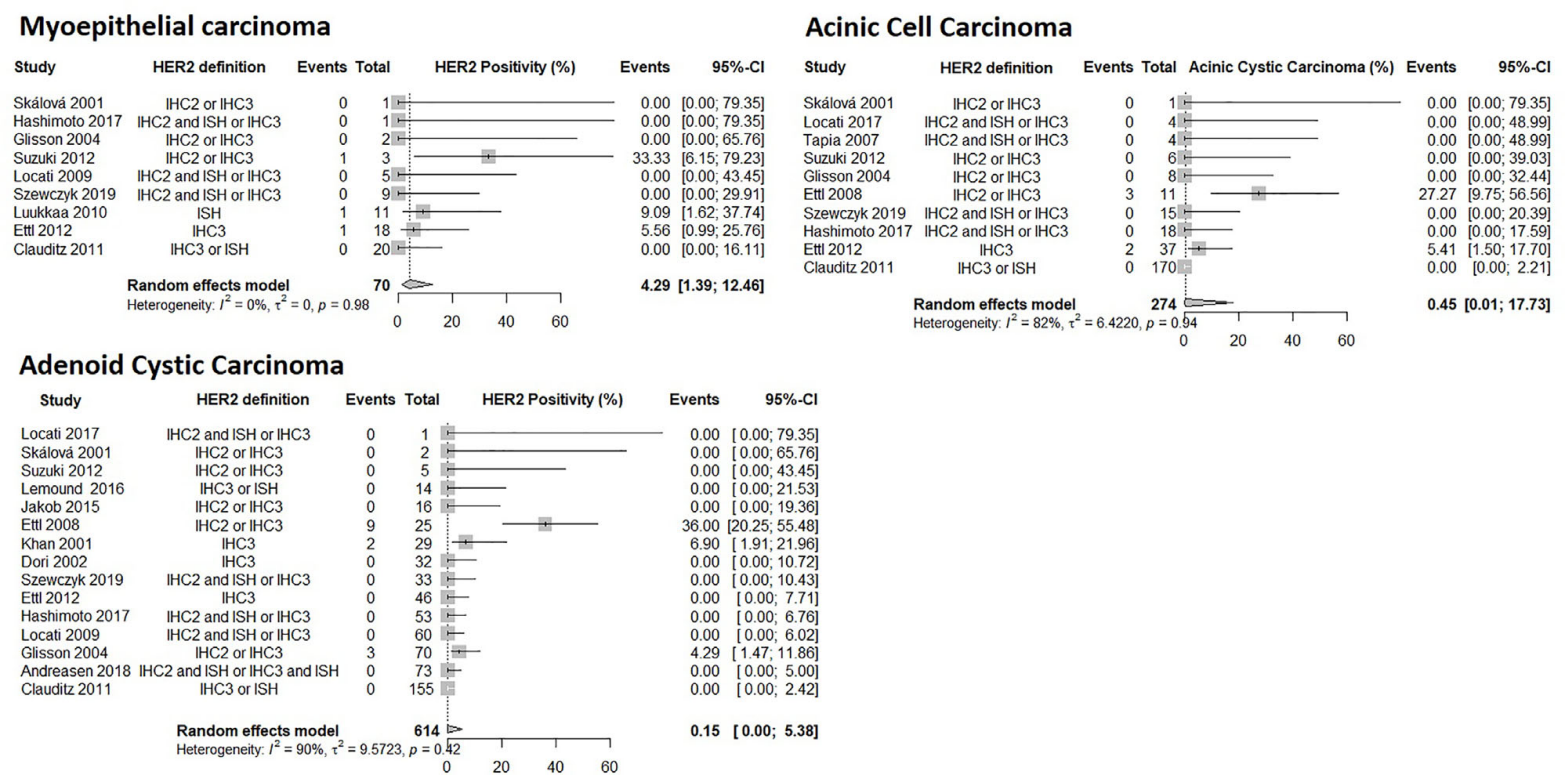
Forrest plot of HER2 Prevalence among SGC subtypes: Myoepithelial carcinoma, Acinic Cell Carcinoma, Adenoid Cystic Carcinoma.

Nine studies were included in the random effects model with a total of 70 patients. The model predicted a prevalence of HER2 positivity in myoepithelial carcinoma patients to be 4.3% (95% CI: 1.4% – 13%) depicted in **Figure 6**. The heterogeneity between studies was not statistically significant.

### Acinic Cell Carcinoma: HER2 Positivity

Ten studies with 274 patients were included in the random effects model. The model predicted a prevalence of HER2 positivity in acinic cell carcinoma patients to be 0.45% (95% CI: 0.0097% – 18%) depicted in **Figure 6**. The heterogeneity between studies was not statistically significant but two studies reported prevalence in the range of 5.4% to 27% while 8 studies reported a prevalence of 0%.

### Adenoid Cystic Carcinoma: HER2 Positivity

Fifteen studies were included in the random effects model with a total of 614 patients. The model predicted the prevalence of HER2 positivity in adenoid cystic carcinoma patients to be 0.15% (95% CI: 0.037% – 5.4%) depicted in **Figure 6**. The heterogeneity between studies was not statistically significant but three studies reported prevalence of 4.3%, 6.9% and 36% while 12 studies reported a prevalence of 0%.

### HER2 Positivity of Other Histological Subtypes

The low number of patients precluded the conduction of meaningful meta-analysis for the following histological subtypes (Full details of studies in **Supplemental S5**): For *epithelial-myoepithelial carcinoma*, 56 patients were included in two studies reporting a single HER2 positive tumor corresponding to a prevalence of 1.8% (95% CI: 0.04%-9.6%).

Seven out of 39 patients with *squamous cell carcinoma* in five studies had HER2 positive tumor corresponding to a prevalence of 17% (95% CI: 7.5%-33%). For *poorly differentiated carcinoma*, 15 patients were included in four studies with one HER2 positive tumor corresponding to 6.7% (95% CI: 0.17%-32%).

One study reported on nine patients with *intraductal carcinoma* with one HER2 positive case corresponding to 11% (95% CI: 0.28% – 48%).

Three studies included 50 patients *with polymorphous adenocarcinoma*, five studies included 33 patients with *basal cell carcinoma*, and three studies included 14 patients with *oncocytic carcinoma*. In all three tumor types, no HER2 positive cases were identified. Two studies reported a total of five patients with *lymphoepithelial carcinoma* of which zero were HER2 positive.

One study reported one patient with *clear cell carcinoma* which was not HER2 positive.

## Discussion

The present work is the first comprehensive meta-analysis providing reliable estimates of the prevalence of HER2 positivity in salivary gland carcinomas including its histological subtypes. The results are summarized in **Tables 2** and **3**. Our results show that salivary gland tumors are very heterogeneous with respect to HER2 positivity ranging from 0% up to 43% with the highest prevalence in SDC which both genomically and morphologically resembles invasive ductal carcinoma of the breast (15). Interestingly, similar frequency measures were seen in histologically related tumors, since both SDC and CEP, as well as epithelial-myoepithelial and myoepithelial carcinoma have comparable estimates. Furthermore, a tendency was noted towards increasing frequency of HER2 positivity in tumor types derived from salivary gland ducts compared to tumors with origin from cells with exocrine function. Accordingly, SDC and SCC displayed high prevalence compared to acinic cell carcinomas and adenoid cystic carcinomas with virtually no HER2 expression. Caution should be advised when evaluating the prevalence estimates of rare histological subtypes with small number of patients and no identified HER2 positive cases.

**Table 2:** Summary of results.

Histological Subtype	Study Included	Number of patients	HER2 positivity estimate (95% CI)
Salivary duct carcinoma	37	1105	43% (95% CI: 36% – 51%)
Carcinoma ex pleomorphic adenoma	14	218	39% (95% CI: 32% – 45%)
Squamous cell carcinoma	5	39	17% (7.5%-33%)
Adenocarcinoma NOS	14	274	13% (7.6% – 21%)
Intraductal carcinoma	1	9	11% (0.28% – 48%)
Poorly differentiated carcinoma	4	15	6.7% (0.17%-32%).
Mucoepidermoid carcinoma	15	591	5.5% (2.9% – 9.6%).
Myoepithelial carcinoma	9	70	4.3% (1.4% – 13%)
Epithelial-myoepithelial carcinoma	2	56	1.8% (0.04%-9.6%)
Acinic cell carcinoma	10	274	0.45% (0.0097% – 18%)
Adenoid cystic carcinoma	14	541	0.15% (0.037% – 5.4%)
Polymorphus adenocarcinoma	3	50	0%
Basal cell carcinoma	5	33	0%
Oncocytic carcinoma	3	14	0%
Lymphoepithelial carcinoma	2	5	0%
Clear cell carcinoma	1	1	0%
Total	50	3372	

**Table 3:** Summary of HER2 protein expression assessed by IHC and HER2 amplification assessed by ISH among SDC patients in the meta-analysis.

Scores of HER2 protein expression	Rate of prevalence (95% CI)	Rate of prevalence of overall HER2 amplification by ISH (95% CI)
IHC0	31% (21-44)	39% (95% CI: 31-49)
IHC1+	10% (6.4-15)	
IHC2+	14% (8.9-20)	
IHC3+	37% (28-47)	

There was sufficient data in four histological subtypes, SDC, CEP, ADC NOS and mucoepidermoid carcinoma to conduct subgroup analyses of the IHC assay used and its correlation with HER2 prevalence. In three of the subgroup analyses: CEP, ADC NOS and mucoepidermoid carcinoma there was no significant difference between the IHC assays used. However, in SDC there was a significant difference based on the IHC assay used, but no difference between probes used in ISH analysis of HER2 amplification (**Figures 2**, **3**). The difference based on IHC assay used may in part be due to inter-observer variability which is thought to be higher when scoring IHC, compared to ISH scoring which is more objective and quantitative (66). Of note, differences disappeared when comparison was restricted to the two most commonly used IHC assays, DAKO and Ventana. There was similarity in frequency measures in IHC and ISH derived estimates of HER2 positivity and amplification of 43% (95% CI: 36% – 51%) and 39% (95% CI: 31-49) respectively.

The criteria used to define HER2 positivity varied among studies with seven different definitions being employed. Subgroup differences between criteria applied to define HER2 positivity were also analyzed (**Figures 4**, **5**). A significant difference depending on the criteria used was observed in ADC NOS and mucoepidermoid carcinoma, in the latter the broadest definition of HER2 positivity of IHC2+ and IHC3+ also yielded the highest prevalence estimate, but this pattern was not as clear in the ADC NOS subgroup analysis. In subtypes with higher prevalence i.e. SDC and CEP subgroup analyses, use of varying criteria did not seem to result in differences in estimated prevalence. Our estimates are limited by these varying criteria for HER2 positivity used in the included studies.

In recent years, it has become common to use IHC2+ confirmed by ISH or IHC3+ as the definition of HER2 positivity as a threshold for using HER2 targeted therapies. In SGC HER2 is often evaluated by use of a HER2 scoring system developed in breast cancer with the use of a threshold chosen based upon clinical response in patients with breast cancer (67).

Another quite unique application of HER2 testing in SGC is its use in the diagnosis of SDC, since this subtype has a higher prevalence of HER2 overexpression and gene amplification than other subtypes.

There is no generally accepted standard treatment of metastatic SGC, and the role of HER2 targeted therapy in this setting is still unclear. Currently there is not sufficient data on newer HER2 targeted drugs in SGC to further define which patient population benefits from the treatment. As such, defining the specific cut-off value to decide which patients should be regarded as “HER2 positive” to receive HER2 targeted therapy remains to be answered. One step in this direction may be the HER2 scoring criteria for SGC proposed by Chatzopoulos et al. (14).

While HER2 treatment results in survival benefits in breast, gastric and esophageal ADC, only limited data are available in SGC. Single agent HER2 directed therapy antitumor effect in patients with HER2 positive SGC is at best modest (7, 8). Several resistance mechanisms have been proposed for HER2 targeted therapy including HER2 receptors lacking extracellular trastuzumab binding domain, upregulation of other tyrosine kinase receptors or alteration of downstream components resulting in aberrant PI3K/Akt/mTOR pathways (68).

But an exact reason to why response with these drugs seem lower in SGC compared to breast cancer and gastric and esophageal ADC has yet to be found. However, HER2 still remains an important potential target for therapies. Thus, promising strategies have emerged applying dual HER2 blockage with trastuzumab and pertuzumab or combining with chemotherapy (trastuzuamb/docetaxel) or as a drug-antibody-conjugate (ado-trastuzumab-emtasine) (9–11).

In summary, the expression of HER2 in SGC is very heterogeneous between and within histological subtypes. The prevalence of HER2 positivity ranged from 0% to 43% in 3,372 patients with sixteen subtypes of SGC. HER2 positivity was most prevalent in SDC and in some tumor subtypes derived from exocrine cells virtually no HER2 expression was reported. Prospective clinical trials are needed to further evaluate novel HER2 directed therapy and to establish the optimal definition of HER2 positivity based on treatment response in SGC with high prevalence of HER2 positivity.

## Data Availability Statement

The original contributions presented in the study are included in the article/**Supplementary Material**. Further inquiries can be directed to the corresponding author.

## Author Contributions

KE and MM-S conceived the project idea. KE and CH reviewed the literature and included studies. KE undertook data analysis. All authors assisted in writing the manuscript and interpreting results. NW, CK, and MM-S provided advice and guidance. All authors contributed to the article and approved the submitted version.

## Conflict of Interest

CK has served in advisory boards for MSD, Bristol-Myers Squibb, and Merck Serono. MM-S has served in advisory boards for Roche, Genmab, Bayer, and Karyopharm Therapeutics and received research grants from Karyopharm Therapeutic, Puma Biotechnologies and MSD.

The remaining authors declare that the research was conducted in the absence of any commercial or financial relationships that could be construed as a potential conflict of interest.
