# Supplementary material for: HER2 Positivity in Histological Subtypes of Salivary Gland Carcinoma: A Systematic Review and Meta-Analysis
Source: Front Oncol. 2021 Jun 24;11:693394. doi: 10.3389/fonc.2021.693394 (PMC8264509; doi:10.3389/fonc.2021.693394)
Supplement: Supplementary file 1 [file DataSheet_1.pdf]

# HER2 positivity in histological subtypes of salivary gland carcinoma: A systematic review and meta-analysis

Kristian Egebjerg MD[1], Cecilie Dupont Harwood MD[2], Nina Claire Woller[3] MD, Claus Andrup Kristensen MD, PhD[1], Morten Mau-Sørensen MD, Ph.D.[1]

[1]Department of Oncology; Copenhagen University Hospital, Rigshospitalet, Copenhagen, Denmark.

[2]Department of Otorhinolaryngology, Head & Neck Surgery and Audiology, Copenhagen, Denmark.

[3]Department of Pathology; Copenhagen University Hospital, Rigshospitalet, Copenhagen, Denmark.

# Supplemental Data

# S1: Overview of included studies and which data was included in meta-analysis

| First Author | Year | Continent | Collection | HER2 Criteria | Probe IHC | ISH_type | Probe ISH | SDC | ADC | CEP | ADCC | ACC | Basal | ClearC | Epi | Poorly | Poly | Mucoep | Myoep | Oncocyt | ClearC | SCC | Lymfo | Intraduct | SDC IHC | SDC ISH |
|---|---|---|---|---|---|---|---|---|---|---|---|---|---|---|---|---|---|---|---|---|---|---|---|---|---|---|
| Khan | 2001 | America | Archival | IHC3 | Novacastra | N/A | N/A | - | - | - | Yes | - | - | - | - | - | - | - | - | - | - | - | - | - | - | - |
| Skálová | 2001 | Europe | Archival | IHC2 or IHC3 | DAKO | N/A | N/A | Yes | Yes | Yes | Yes | Yes | Yes | - | - | Yes | - | Yes | Yes | - | - | - | - | - | - | - |
| Dori | 2002 | Asia | Archival | IHC3 | DAKO | N/A | N/A | - | - | - | Yes | - | - | - | - | - | - | - | - | - | - | - | - | - | - | - |
| Skalova | 2003 | Europe | Archival | IHC2 and ISH or IHC3 | DAKO | FISH | Ventana | Yes | - | - | - | - | - | - | - | - | - | - | - | - | - | - | - | - | Yes | Yes |
| Glisson | 2004 | America | Archival | IHC2 or IHC3 | DAKO | N/A | N/A | Yes | Yes | Yes | Yes | Yes | - | - | - | | - | Yes | Yes | - | - | Yes | - | - | Yes | - |
| Weed | 2004 | America | Archival | IHC2 or IHC3 | DAKO | N/A | N/A | - | - | - | - | - | - | - | - | | - | Yes | - | - | - | - | - | - | - | - |
| Di Palma | 2005 | Europe | Archival | IHC3 | Neomarkers | FISH | PathVysion | - | | Yes | - | - | - | - | - | - | - | - | - | - | - | - | - | - | - | - |
| Jaehne | 2005 | Europe | Archival | IHC3 | DAKO | N/A | N/A | Yes | - | - | - | - | - | - | - | - | - | - | - | - | - | - | - | - | Yes | - |
| Cornolti | 2007 | Europe | Archival | IHC3 or ISH | DAKO | FISH | PathVysion | Yes | - | - | - | - | - | - | - | - | - | - | - | - | - | - | - | - | Yes | Yes |
| Nabili | 2007 | America | Archival | IHC3 | DAKO | FISH | PathVysion | Yes | - | - | - | - | - | - | - | - | - | - | - | - | - | - | - | - | - | Yes |
| Tapia | 2007 | Europe | Na | IHC2 and ISH or IHC3 | DAKO | FISH | PathVysion | - | Yes | - | - | Yes | - | - | - | - | - | Yes | | - | - | Yes | - | - | - | - |
| Williams | 2007 | America | Archival | IHC3 | Neomarkers | N/A | N/A | Yes | - | - | - | - | - | - | - | - | - | - | - | - | - | - | - | - | Yes | - |
| Ettl | 2008 | Europe | Archival | IHC2 or IHC3 | DAKO | N/A | N/A | Yes | Yes | Yes | Yes | Yes | Yes | - | - | - | - | Yes | | Yes | - | Yes | Yes | - | - | - |
| Shang | 2008 | Asia | Archival | IHC2 or IHC3 | DAKO | N/A | N/A | - | - | - | - | - | - | - | - | - | - | Yes | - | - | - | - | - | - | - | - |
| Locati | 2009 | Europe | Archival | IHC2 and ISH or IHC3 | DAKO | N/A | PathVysion | Yes | Yes | - | Yes | - | - | - | - | Yes | - | Yes | Yes | - | - | - | - | - | - | Yes |
| Luukkaa | 2010 | Finland and Canada | Archival | ISH | Ventana | SISH | Ventana | - | - | - | - | - | - | - | - | - | - | | Yes | - | - | - | - | - | - | - |
| Williams | 2010 | America | Archival | IHC2 and ISH or IHC3 | Labvision | FISH | PathVysion | Yes | - | - | - | - | - | - | - | - | - | - | - | - | - | - | - | - | Yes | Yes |
| Clauditz | 2011 | Europe | Archival | IHC3 or ISH | DAKO | FISH | PathVysion | Yes | Yes | - | Yes | Yes | Yes | - | Yes | - | Yes | Yes | Yes | Yes | - | | - | - | Yes | Yes |
| Di Palma | 2012 | Europe | Archival | IHC2 and ISH or IHC3 | DAKO | SISH | Ventana | Yes | - | - | - | - | - | - | - | - | - | - | - | - | - | - | - | - | Yes | Yes |

| | | | | | | | | | | | | | | | | | | | | | | | | | | |
|---|---|---|---|---|---|---|---|---|---|---|---|---|---|---|---|---|---|---|---|---|---|---|---|---|---|---|
| Ettl | 2012 | Europe | Archival | IHC3 | DAKO | FISH | ZytoVision | Yes | Yes | - | Yes | Yes | - | - | - | - | Yes | Yes | Yes | - | - | Yes | - | - | - | Yes |
| Hashimoto | 2012 | Asia | Archival | IHC2 and ISH or IHC3 | DAKO | CISH | Invitrogen | - | - | Yes | - | - | - | - | - | - | - | - | - | - | - | - | - | - | - | - |
| Suzuki | 2012 | Asia | Archival | IHC2 or IHC3 | DAKO | FISH | ABOTT | Yes | Yes | Yes | Yes | Yes | Yes | - | | - | | Yes | Yes | - | - | | Yes | - | - | Yes |
| Cros | 2013 | Europe | Archival | IHC3 | DAKO | N/A | N/A | Yes | Yes | Yes | - | - | - | - | - | - | - | - | - | - | - | - | - | - | - | - |
| Nakano | 2013 | Asia | Archival | IHC2 or IHC3 | DAKO | CISH | DAKO | - | - | - | - | - | - | - | - | - | - | Yes | - | - | - | - | - | - | - | - |
| Nardi | 2013 | America | Archival | ISH | No IHC done | FISH | PathVysion | Yes | - | - | - | - | - | - | - | - | - | - | - | - | - | - | - | - | - | Yes |
| Kondo | 2014 | Asia | Archival | IHC2 and ISH or IHC3 | Pathway | DISH | Ventana | Yes | - | Yes | - | - | - | - | - | - | - | - | - | - | - | - | - | - | Yes | Yes |
| Masubuchi | 2014 | Asia | Archival | ICH3 and ISH | Pathway | FISH | DAKO | Yes | - | - | - | - | - | - | - | - | - | - | - | - | - | - | - | - | - | Yes |
| Han | 2015 | Asia | Archival | IHC2 and ISH or IHC3 | DAKO | FISH | Abott | Yes | - | - | - | - | - | - | - | - | - | - | - | - | - | - | - | - | Yes | - |
| Jakob | 2015 | America | Archival | IHC2 or IHC3 | Labvision | N/A | N/A | - | - | - | Yes | - | - | - | - | - | - | - | - | - | - | - | - | - | - | - |
| Locati | 2016 | Europe | Archival | IHC2 and ISH or IHC3 | DAKO | FISH | Abott | Yes | Yes | - | | - | | - | | Yes | - | | - | - | - | | - | - | - | - |
| Nishijima | 2015 | Asia | Archival | IHC2 or IHC3 | DAKO | CISH | DAKO | - | - | Yes | | - | | - | | | - | | - | - | - | | - | - | - | - |
| Kusafuka | 2016 | Asia | Archival | IHC2 and ISH or IHC3 | DAKO | FISH | PathVysion | Yes | - | - | - | - | - | - | - | - | - | - | - | - | - | - | - | - | Yes | Yes |
| Lemound | 2016 | Europe | Archival | IHC3 or ISH | Ventana | CISH | Zytomed | Yes | Yes | Yes | Yes | - | - | - | - | - | - | Yes | - | - | - | | - | - | - | - |
| Luk | 2016 | Oceania | Archival | IHC2 and ISH or IHC3 | Ventana | FISH | PathVysion | Yes | - | - | - | - | - | - | - | - | - | - | - | - | - | - | - | - | Yes | Yes |
| Hashimoto | 2017 | Asia | Archival | IHC2 and ISH or IHC3 | Ventana | N/A | N/A | Yes | Yes | Yes | Yes | Yes | Yes | Yes | Yes | Yes | Yes | Yes | Yes | Yes | Yes | Yes | - | - | - | - |
| Khoo | 2017 | Oceania | Archival | ISH | Ventana | FISH | PathVysion | Yes | - | - | - | - | - | - | - | - | - | - | - | - | - | - | - | - | - | Yes |
| Locati | 2017 | Europe | Archival | IHC2 and ISH or IHC3 | DAKO | N/A | N/A | - | Yes | Yes | Yes | Yes | - | - | - | - | - | Yes | - | - | - | - | - | - | - | - |
| Takase | 2017 | Asia | Archival | ICH3 or ISH | DAKO | FISH | DAKO | Yes | - | - | - | - | - | - | - | - | - | - | - | - | - | - | - | - | - | - |
| Andreasen | 2018 | Europe | Archival | IHC2 and ISH or IHC3 and ISH | Roche | FISH | NA | - | - | - | Yes | - | - | - | - | - | - | - | - | - | - | - | - | - | - | - |
| Boon | 2018 | Europe | Archival | IHC2 and ISH or IHC3 | DAKO | FISH | Kreatech | Yes | - | - | - | - | - | - | - | - | - | - | - | - | - | - | - | - | - | - |
| Beck | 2018 | Europe | Archival | IHC3 | Ventana | N/A | N/A | Yes | | Yes | | - | - | - | - | - | - | - | - | - | - | - | - | - | Yes | - |
| Kanazawa | 2018 | Asia | Archival | IHC3 | DAKO | N/A | N/A | Yes | - | - | - | - | - | - | - | - | - | - | - | - | - | - | - | - | - | - |

| | | | | | | | | | | | | | | | | | | | | | | | | | | |
|---|---|---|---|---|---|---|---|---|---|---|---|---|---|---|---|---|---|---|---|---|---|---|---|---|---|---|
| Ryu | 2018 | Asia | Archival | ISH | Ventana | SISH | Ventana | Yes | - | - | - | - | - | - | - | - | - | - | - | - | - | - | - | - | Yes | Yes |
| Gargano | 2019 | America | Archival | IHC3 | N/A | N/A | N/A | Yes | - | - | - | - | - | - | - | - | - | - | - | - | - | - | - | - | - | - |
| Liang | 2019 | America | Archival | IHC3 | Ventana | N/A | N/A | Yes | Yes | Yes | - | - | - | - | - | - | - | - | - | - | - | - | - | - | - | - |
| Santana | 2019 | Europe | Archival | IHC2 and ISH or IHC3 | Ventana | FISH | PathVysion | Yes | | Yes | - | - | - | - | - | - | - | - | - | - | - | - | - | - | Yes | Yes |
| Szewczyk | 2019 | Europe | Archival | IHC2 and ISH or IHC3 | Roche | N/A | N/A | Yes | Yes | Yes | Yes | Yes | - | - | - | - | - | Yes | Yes | - | - | - | - | - | - | - |
| Villepelet | 2019 | Europe | Prospective | IHC2 and ISH or IHC3 | Ventana | N/A | N/A | Yes | - | - | - | - | - | - | - | - | - | - | - | - | - | - | - | - | Yes | - |
| Chatzopoulos | 2020 | America | Archival | IHC2 and ISH or IHC3 | Ventana | FISH | PathVysion | Yes | - | - | - | - | - | - | - | - | - | - | - | - | - | - | - | - | Yes | Yes |
| Hsieh | 2020 | Europe | Archival | IHC2 and ISH or IHC3 | Ventana | FISH | Zytovision | Yes | - | - | - | - | - | - | - | - | - | - | - | - | - | - | - | Yes | Yes | Yes |

SDC = Salivary Duct Carcinoma, ADC= Adenocarcinoma not otherwise specified, CEP = Carcinoma ex pleomorphic adenoma, ADCC = Adenoid cystic carcinoma, ACC= Acinic cell carcinoma, Basal= Basal cell adenocarcinoma, ClearC = Clear cell carcinoma, Epi = Epithelial myoepithelial carcinoma, Poorly= Poorly differentiated carcinoma positive Poly= Polymorphus adenocarcinoma, Mucoep= Mucoepidermoid carcinoma positive, Mycoep= Myoepithelial carcinoma positive, Oncocyt= Oncocytic carcinoma positive, SCC= Squamous cell carcinoma positive, Lymfo = Lymfoepithelial carcinoma positive, Intraduct = Intraductal Carcinoma Positive

# S2: Search syntax

Searched up to 19th September 2020

**PUBMED:**

((salivary gland tumor[Title/Abstract] OR carcinoma of the salivary gland[Title/Abstract] OR salivary gland cancer[Title/Abstract])) AND (HER2 or c-ERB2)

Results: 37 hits

**Web of science:**

AB=(salivary gland tumor  OR carcinoma of the salivary gland OR salivary gland cancer) AND AB=(HER2  OR c-erB2)

Results:  100 hits

**EMBASE:**

((salivary gland tumor or carcinoma of the salivary gland or salivary gland cancer).ab and HER2.af) or c-ERB2 af.

Results: 95 hits

# S3: Full list of texts screened but not included (n=74)

| First Author | Year | PMID | Reason for exclusion |
|---|---|---|---|
| Yamada | 1989 | 2508310 | No HER2 data |
| Kernohan | 1991 | 1672154 | No quantitative SGC HER2 IHC or ISH data |
| Shrestha | 1992 | 1361005 | No quantitative SGC HER2 IHC or ISH data |
| Sugano | 1992 | 1353853 | “Unique” scoring system of HER2 IHC |
| Delgado | 1993 | 7688652 | No quantitative SGC HER2 IHC or ISH data |
| Hellquist | 1994 | 7931825 | No quantitative SGC HER2 IHC or ISH data |
| Kärjä | 1994 | 7915830 | No quantitative SGC HER2 IHC or ISH data |
| Müller | 1994 | 7991521 | No quantitative SGC HER2 IHC or ISH data |
| Press | 1994 | 7522962 | No quantitative SGC HER2 IHC or ISH data |
| Cho | 1995 | 7726139 | No quantitative SGC HER2 IHC or ISH data |
| Giannoni | 1995 | 7870438 | No quantitative SGC HER2 IHC or ISH data |
| Shintani | 1995 | 8669836 | No quantitative SGC HER2 IHC or ISH data |
| Felix | 1996 | 8666365 | No quantitative SGC HER2 IHC or ISH data |
| Kamio | 1996 | 8925128 | No quantitative SGC HER2 IHC or ISH data |
| Rosa | 1996 | 8729044 | No quantitative SGC HER2 IHC or ISH data |
| Cho | 1997 | 9443087 | Case report and no quantitative SGC HER2 IHC or ISH data |

| | | | |
|---|---|---|---|
| Martinez-Barba | 1997 | 8625227 | (Listed as review) No quantitative SGC HER2 IHC or ISH data |
| Suzuki | 1998 | 10211217 | No quantitative SGC HER2 IHC or ISH data |
| Wick | 1998 | 9426521 | No quantitative SGC HER2 IHC or ISH data |
| Cho | 1999 | 10402521 | No quantitative SGC HER2 IHC or ISH data |
| Jaehne | 2001 | 11555785 | Article in German |
| Gibbons | 2001 | 11568571 | No quantitative SGC HER2 IHC or ISH data |
| Lewis | 2001 | 11431714 | No quantitative SGC HER2 IHC or ISH data |
| O'Malley | 2001 | 11293897 | Study on breast cancer, no SGC HER2 IHC or ISH data |
| Haddad | 2003 | 12907212 | Preselected patient population |
| Nguyen | 2003 | 14974865 | Not accessible |
| Nagler | 2003 | 12759537 | No prevalence data |
| Etges | 2003 | 14645349 | No quantitative SGC HER2 IHC or ISH data |
| Simpson | 2003 | 12883239 | Case report |
| Brandwein-Gensler | 2004 | 15252310 | Case report and no definition of HER2 |
| Dagrada | 2004 | 14987238 | Article is a case report / "comment" |
| Nagao | 2004 | 15323139 | Case report |
| Freitas | 2005 | 16273189 | No quantitative SGC HER2 IHC or ISH data |

| | | | |
|---|---|---|---|
| Locati | 2005 | 7991521 | Article is a “Comment” |
| Laurie | 2006 | 16763282 | Review |
| Sørensen | 2006 | 16630292 | No data on HER2 |
| Agulnik | 2007 | 17761983 | Study on pre-selected population |
| Ihrler | 2007 | 17593217 | No data on HER2 |
| Matsubayashi | 2007 | 17310348 | No quantitative IHC or ISH HER2 data |
| Sasaki | 2007 | 17192791 | No data on HER2 status in SGC |
| Johnson | 2008 | 18091319 | Not accessible |
| Ettl | 2008 | 18214402 | Article in German |
| Nashed | 2009 | 18405406 | Case report |
| Vidal | 2009 | 19309723 | Study on pre-selected population |
| Lujan | 2010 | 20664595 | No HER2 data |
| Hashimoto | 2011 | 21317706 | Data already included in Hashimoto 2012 |
| Santini | 2012 | 24006824 | Article in French |
| Ettl | 2012 | 22240798 | No discrimination between histological subtype and HER2 status |
| Golusinski | 2013 | 24021827 | Article in Polish |
| Jayaprakash | 2014 | 23606370 | No HER2 data |

| | | | |
|---|---|---|---|
| Limaye | 2013 | 23429737 | Study on pre-selected population |
| Ettl | 2013 | 23780687 | No discrimination between histological subtype and HER2 status |
| Kuo | 2013 | 23821212 | Review |
| Otsuka | 2013 | 24191589 | Article in Japanese |
| Perissinotti | 2013 | 23749912 | Study on pre-selected population |
| Simpson | 2013 | 23821208 | Review |
| Stenman | 2013 | 23821214 | Review |
| Yamamoto | 2014 | 25087467 | Case Report |
| Grünewald | 2015 | 26053092 | No IHC or ISH HER2 data |
| Huo | 2015 | 26575266 | No SGC data |
| Masubuchi | 2015 | 24553861 | Does not discriminate between SGC subtypes |
| Dalin | 2016 | 27103403 | No extractable HER2 IHC data |
| Gilbert | 2016 | 26939990 | Study not clear HER2 positivity criteria |
| Wang | 2016 | 27334835 | No quantitative SGC HER2 IHC or ISH data |
| Krings | 2017 | 28548128 | HER2 status not performed |
| Schmitt | 2017 | 29103750 | Review |
| Shams | 2018 | N/A | Article not accessible |
| Takahashi | 2018 | 30452336 | Study on pre-selected population |

| | | | |
|---|---|---|---|
| Kurzrock | 2019 | 32067683 | Study on pre-selected population |
| Stodulski | 2019 | 31062093 | No definition of HER2 positivity |
| Hanna | 2020 | 32310325 | No discrimination between histological subtype and HER2 status |

# S4: Salivary Duct Carcinoma overall and IHC assay subgroup analysis

| Salivary duct carcinoma: Overall | | | |
|---|---|---|---|
| **Assay** | **Number of studies** | **Prevalence Estimate** | **95% CI** |
| DAKO | 19 | 46.4340 | 31.7966; 61.7127 |
| Labvision | 1 | 15.1515 | 8.3507; 25.9243 |
| Neomarkers | 1 | 27.1186 | 17.3278; 39.7797 |
| No IHC done | 1 | 42.1053 | 22.6327; 64.3884 |
| Roche | 3 | 46.1538 | 20.5701; 73.9378 |
| Unknown | 1 | 39.2857 | 23.2592; 58.0079 |
| Ventana | 11 | 44.1469 | 35.6145; 53.0397 |
| Test for subgroup differences (random effects model): p-value: 0.0017 | | | |

| Salivary Duct Carcinoma: IHC 0 | | | |
|---|---|---|---|
| **Assay** | **Number of studies** | **Prevalence Estimate** | **95% CI** |
| Ventana | 8 | 25.1282 | 18.5098; 33.1504 |
| DAKO | 8 | 32.6954 | 11.0481; 65.5170 |
| Neomarkers | 1 | 26.1905 | 17.9097; 36.5933 |
| Labvision | 1 | 74.2424 | 62.4083; 83.3453 |
| Test for subgroup differences (random effects model): p-value < 0.0001 | | | |

| Salivary Duct Carcinoma: IHC 1+ | | | |
|---|---|---|---|
| **Assay** | **Number of studies** | **Prevalence Estimate** | **95% CI** |
| Ventana | 8 | 14.0028 | 7.5018; 24.6370 |
| DAKO | 8 | 5.4824 | 1.6132; 17.0252 |
| Neomarkers | 1 | 19.0476 | 12.0097; 28.8573 |
| Labvision | 1 | 6.0606 | 2.2934; 15.0620 |
| Test for subgroup differences (random effects model): p-value 0.0361 | | | |

| Salivary Duct Carcinoma: IHC2+ | | | |
|---|---|---|---|
| **Assay** | **Number of studies** | **Prevalence Estimate** | **95% CI** |
| Ventana | 8 | 18.9744 | 13.1982; 26.5064 |
| DAKO | 8 | 7.7966 | 1.9964; 25.9814 |
| Neomarkers | 1 | 29.7619 | 20.9757; 40.3492 |
| Labvision | 1 | 4.5455 | 1.4734; 13.1666 |
| Test for subgroup differences (random effects model): p-value 0.0011 | | | |

| Salivary Duct Carcinoma: IHC3+ | | | |
|---|---|---|---|
| **Assay** | **Number of studies** | **Prevalence Estimate** | **95% CI** |
| Ventana | 8 | 40.5947 | 30.8457; 51.1461 |
| DAKO | 8 | 41.4235 | 21.1502; 65.0884 |
| Neomarkers | 1 | 25.0000 | 16.9036; 35.3258 |
| Labvision | 1 | 15.1515 | 8.3507; 25.9243 |
| Test for subgroup differences (random effects model): p-value 0.0017 | | | |

# S5: Tissue types with low number of observations.

## Squamous Cell Carcinoma

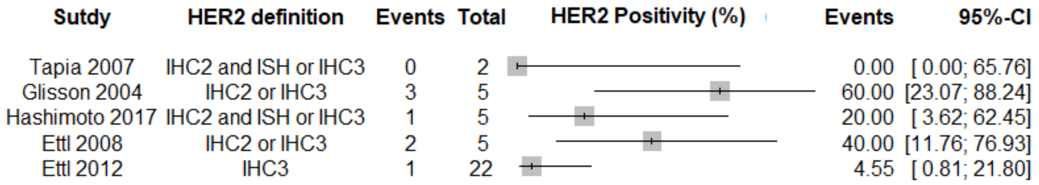


## Epithelial-myoepithelial carcinoma

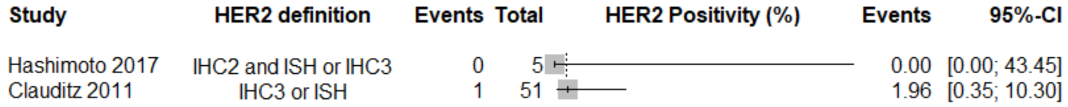


## Poorly differentiated carcinoma

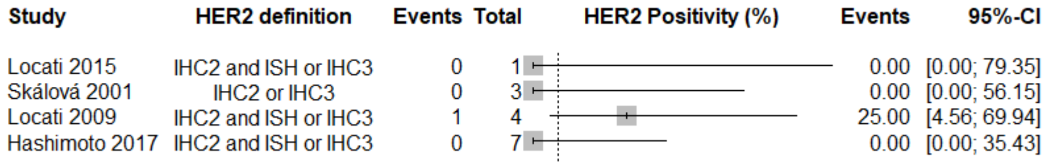


## Polymorphus adenocarcinoma

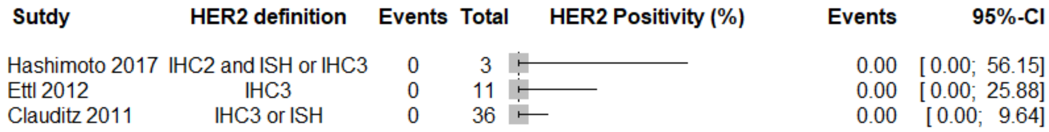


## Basal Cell Carcinoma

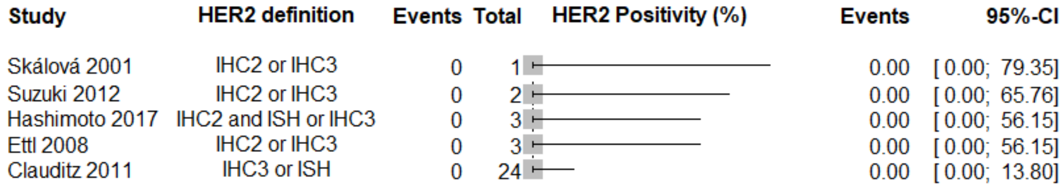


## Oncocytic Carcinoma

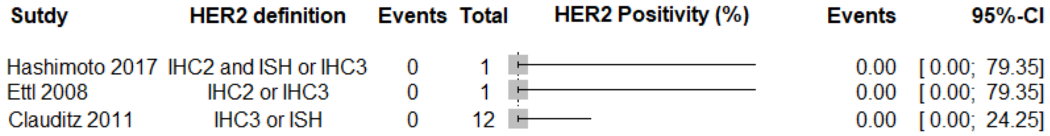


Hashimoto 2017 reported one patient with clear cell carcinoma with no HER2 positivity defined as IHC2+ and ISH amplification or IHC3+.

Suzuki 2012 and Ettl 2008 reported a total of five patients with lymphoepithelial carcinoma of which zero were HER2 positive.

Hsieh 2020 reported on nine patients of Intraductal Carcinoma in which one was IHC2+.
